# Effects of Particle Size on the Structure and Photocatalytic Performance by Alkali-Treated TiO_2_

**DOI:** 10.3390/nano10030546

**Published:** 2020-03-18

**Authors:** Danqi Li, Hongchen Song, Xia Meng, Tingting Shen, Jing Sun, Wenjia Han, Xikui Wang

**Affiliations:** 1 State Key Laboratory of Biobased Material and Green Papermaking, School of Environmental Science and Engineering, Qilu University of Technology (Shandong Academy of Sciences), Jinan 250353, China; autantenporte@163.com (D.L.); shc01261005lx@gmail.com (H.S.); mengxia7184@126.com (X.M.); stthunanyt@163.com (T.S.); hwj200506@163.com (W.H.); 2 College of Environmental Science and Engineering, Shandong Agriculture and Engineering University, Jinan 251100, China

**Keywords:** TiO_2_, alkali, particle size, surface oxygen defects, photocatalysis

## Abstract

Particle size of nanomaterials has significant impact on their photocatalyst properties. In this paper, TiO_2_ nanoparticles with different crystalline sizes were prepared by adjusting the alkali-hydrothermal time (0–48 h). An annealing in N_2_ atmosphere after hydrothermal treatment caused TiO_2_ reduction and created defects, resulting in the visible light photocatalytic activity. The evolution of physicochemical properties along with the increase of hydrothermal time at a low alkali concentration has been revealed. Compared with other TiO_2_ samples, TiO_2_-24 showed higher photocatalytic activity toward degrading Rhodamine B and Sulfadiazine under visible light. The radical trapping and ESR experiments revealed that O_2_^•-^ is the main reactive specie in TiO_2_-24. Large specific surface areas and rapid transfer of photogenerated electrons are responsible for enhancing photocatalytic activity. The above findings clearly demonstrate that particle size and surface oxygen defects can be regulated by alkali-hydrothermal method. This research will deepen the understanding of particle size on the nanomaterials performance and provide new ideas for designing efficient photocatalysts.

## 1. Introduction

Titanium dioxide (TiO_2_) has been the most widely used multifunctional material due to its low cost, non-toxicity, wide source, acid-base resistance and high catalytic activity [1]. The outstanding features of TiO_2_ greatly extends its application from traditional areas to recent promising fields such as photocatalysis, water splitting, lithium batteries and recently emerging perovskite solar cells [2,3]. However, large band gap of TiO_2_ and high electron hole recombination limit its photocatalytic performance [4]. To modify these intrinsic drawbacks, tremendous efforts have been devoted to broadening the active spectrum and promoting the charge transport [5,6].

Particle size is a crucial factor affecting the performance of nano-photocatalytic materials. The size and shape of the catalyst influence its surface structure and then resulting in various catalytic performance [7,8]. Compared with bulk TiO_2_, granular TiO_2_ nanoparticles have a large surface area and a broadened band gap. They contain more active sites and display improved photocatalytic activity, photoelectrochemical properties and gas sensitivity [9,10]. Lin et al. successfully synthesized TiO_2_ nanoparticles of different particle sizes (12–29 nm) and found the band gap of the TiO_2_ nanoparticles was a function of the primary particle size. The results also showed that the primary particle size is closely related to the electro-optical properties of photocatalysts. As the primary particle size increases, the photocatalytic rate constant decreases exponentially [11]. Cheng et al. found that the AgI/BiOI photocatalysts display size-dependent photocatalytic activity, which increases with the smaller size of the AgI NPs. This is believed to be related to the larger number of surface active sites and faster spatial charge transfer [12]. Kocı et al. have proved that as the particle size changed, the specific surface area, charge-carrier dynamics and light absorption efficiency of TiO_2_ nanoparticles also changed, and then the yield changed accordingly [13]. Qi et al. summarized that the antimicrobial activity of ZnO NPs exhibits size dependency, that is, small ZnO NPs yield high antibacterial activities [14]. These researches have confirmed that the particle size has an important effect on the catalyst properties.

There are many methods that have been developed to prepare TiO_2_ nanostructures with various morphology types and sizes, including the hydrothermal method, the sol-gel method, the vapor deposition method and the electrospinning method [9,15]. Nowadays the hydrothermal synthesis of TiO_2_ nanostructures is a well-established technique and typically yields a large amount of TiO_2_ nanostructures from solution [16,17]. Most of the current researches focus on the alkali-hydrothermal approaches with high alkali concentration (8–12 M), which usually produces different morphology types of 1D TiO_2_ nanostructures (nanotube, nanorod, nanowire, nanofiber or nanobelt) [18,19]. Although the properties and applications of TiO_2_ and nanostructures has been studied, the effect of alkali on TiO_2_ morphology types, sizes and photocatalytic performance is still unavailable. The effect of low alkali concentration on TiO_2_ is less studied. 

In this study, we aimed to provide a comprehensive understanding of the effects of low alkali-hydrothermal treatment on the TiO_2_ structure and photocatalytic performance. TiO_2_ nanoparticles with different particle sizes were prepared by adjusting hydrothermal time by the same preparation method. The effects of particle size on photocatalytic activity and photocarriers migration rate were systematically compared. This research emphasizes the important role of particle size in regulating the photocatalyst structure and performance.

## 2. Materials and Methods 

### 2.1. Chemicals

Anatase titanium oxide (CAS: 13463-67-7) was purchased from Sigma Aldrich (St Louis, MO, USA) and Sulfadiazine (SD) was purchased from Aladdin (Shanghai, China). All other chemicals were analytical reagent grade and used as received.

### 2.2. Preparation of TiO_2_ Samples

The preparation of TiO_2_-X photocatalysts was performed by a modified alkali-hydrothermal method [20]. Alkali ions penetrate into the TiO_2_ lattice, or combine with oxygen to form alkali metal oxides on the TiO_2_ surface, which limit the growth of TiO_2_ nanocrystals. Therefore, the alkali-hydrothermal method is used to adjust the size of titanium dioxide particles. Generally, 0.6 g Anatase TiO_2_, 60 mL D.I. water and 12 g NaOH were added into a beaker with stirring for 30 min. The mixture was transferred to a 100 mL Teflon-lined stainless steel autoclave and heated to 180 °C with different time. The obtained sediment was washed several times with D.I. water and HCl solution (0.6 M) to adjust the pH to remain neutral. The samples were freeze-dried and then calcined at 400 °C for 2 hours under N_2_ atmosphere. For the sake of distinction, we define the sample obtained by hydrothermal treatment for X hours as TiO_2_-X.

### 2.3. Characterization 

The crystalline structure of the prepared TiO_2_ nanomaterial was characterized by X-ray diffraction (XRD, D8 ADVANCE, Bruker AXS, Karlsruhe, Germany) with the scan range from 2° to 80°. The morphology of photocatalysts was observed by transmission electron microscope (TEM, JEM-2100F, JEOL, Tokyo, Japan). Elemental analysis in the material was characterized by X-ray photoelectron spectroscopy (XPS, ESCALAB Xi^+^, Thermo Fisher Scientific, Waltham, MA, USA) and Raman spectra (Raman, Senterra R200-L, Bruker Optics, Karlsruhe, Germany) with a 532 nm laser beam as the excitation source. N_2_ adsorption-desorption isotherms were obtained using the Micromeritics (Gemini V2380, Micromeritics, Norcross, GA, USA). The optical absorption properties of photocatalysts were tested by the ultraviolet-visible diffuse reflectance spectroscopy (DRS, Hitachi U-300, Hitachi, Tokyo, Japan) with a scanning range of 200–800 nm. The electron spin resonance (ESR, A300, Bruker, Karlsruhe, Germany) spin-trap technique was employed to monitor the reactive oxygen radical species generated during the irradiation with spin-trap reagent 5,5-dimethyl-1-pirroline-N-oxide (DMPO). Photoluminescence (PL, Hitachi F-4500, Hitachi, Tokyo, Japan) spectra were measured with a continuous wave laser (325 nm) as the exciting source. The main oxidative species in the photocatalytic process could be detected through the free radical trapping experiment by using t-BuOH (hydroxyl radical scavenger), EDTA-2Na (hole scavenger) and circulating N_2_ (superoxide radical scavenger).

### 2.4. Photocatalytic Experiments

The photocatalysis was carried out using a 300 W Xe arc lamp (PLS-SXE 300, Perfectlight Co. Ltd., Beijing, China) equipped with a UV-cutoff filter (λ > 400 nm). RhB (Rhodamine B) and SD (Sulfadiazine) were selected as organic contaminants. 0.15 g photocatalyst was placed in 150 mL 20 ppm RhB or 150 mL 10 ppm SD aqueous solution and stirred in the dark to reach an adsorption-desorption equilibrium before illumination. During the period of visible light illumination, the temperature was kept at 20 °C by recirculating cooling water. A 5 mL aliquot was taken at 15 min intervals and filtered through a 0.45 micron filter. The residual concentration of RhB was estimated by visible spectrophotometer (722N, Shanghai Precision & Scientific Instrument Co. Ltd., Beijing, China). The concentration of SD was measured by high performance liquid chromatography (LC-20AT, Shimadzu, Kyoto, Japan) with a C18 (4.6 × 250) reversed phase column. The wavelength of photodiode array detector was 269 nm for SD. The mobile phase was selected as acetonitrile and water (V:V = 1:3) at a flow rate of 0.6 mL/min. 

## 3. Results

### 3.1. Characterization of Prepared TiO_2_-X

**Figure 1:**
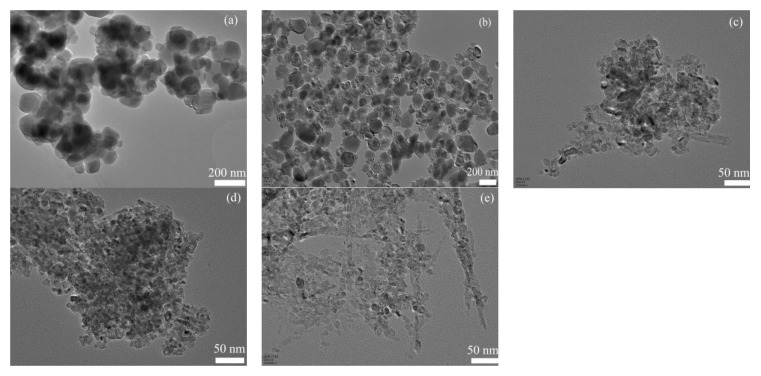
Transmission electron microscopy images of (**a**) TiO_2_, (**b**) TiO_2_-3, (**c**) TiO_2_-12, (**d**) TiO_2_-24, (**e**) TiO_2_-48.

Figure 1 shows the TEM images of pristine TiO_2_ and prepared TiO_2_ with different hydrothermal times (TiO_2_-X). The particle diameters of pristine TiO_2_ are distributed in the range from 50 to 250 nm. After hydrothermal treatment for 3 h (Figure 1b), the TiO_2_ particles show clear grain boundaries and agglomerations. The particle size decreases obviously to an average of 100 nm and a small amount of particles about 20 nm can be seen. TiO_2_-12 (Figure 1c) exhibits an irregular morphology. Most of the crystal decreased to 10–40 nm and stacked together, which might indicate the raw materials begin to dissolve, decompose and nucleate. TiO_2_-24 is composed of 10–20 nm nanoparticles. As for TiO_2_-48, TiO_2_ nanocrystals experience a growth process along a certain crystallographic orientation to form a rod-like structure.

**Figure 2:**
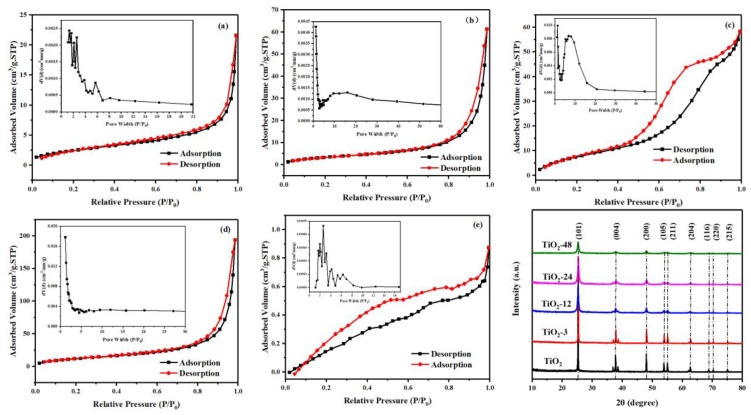
N_2_ adsorption-desorption isotherm of (**a**) pristine TiO_2_, (**b**) TiO_2_-3, (**c**) TiO_2_-12, (**d**) TiO_2_-24, (**e**) TiO_2_-48. Inset figures are the corresponding pore size distribution. (**f**) XRD patterns of TiO_2_, TiO_2_-3, TiO_2_-12, TiO_2_-24, TiO_2_-48.

The porous structure and specific surface areas of TiO_2_-X were characterized by nitrogen adsorption-desorption isotherms as well as the pore size distributions in Figure 2. All samples exhibit type IV nitrogen isotherm with a hysteresis loop, indicating the mesopores features [21]. The hysteresis loops of pristine TiO_2_, TiO_2_-3, TiO_2_-24 pertain to H3-type, that is caused by the unsaturated adsorption of nitrogen in relatively high P/P_0_ [22,23]. For TiO_2_-12, a hysteresis loop is observed with distinct saturated adsorption platform in a wide relative pressure region. That belongs to the H2-type and reflects a relatively complex pore structure, which is related to its irregular morphology and stacking between layers in figure TEM [24,25]. TiO_2_-48 shows H2-type hysteresis loops distributed in the whole relative pressure region, mainly due to the irregular morphology and uneven pore size distribution caused by the gradual transformation of nanoparticles into nanorods [26]. The specific surface area, pore size and pore volume data are listed in Table 1 It is indicated that the specific surface area and pore volume increases with decreasing particle size. In Table 1, TiO_2_-24 possessed the largest surface areas (78.888 m^2^/g) and pore volume (0.312 cm^3^/g) nearly six times and 10 times larger compared to the pristine TiO_2_ (13.060 m^2^/g and 0.035 cm^3^/g), respectively. For long treatment, particles get more and more aggregated, leading to reduction in surface area and pore volume (growth also inside the pores). 

The X-ray diffraction patterns recorded from TiO_2_ with different hydrothermal treatment time are shown in Figure 2f. All the relatively sharp peaks could be indexed as anatase TiO_2_ which are basically in agreement with the anatase phase (JCPDS No. 84-1285). Firstly, the intensity of the diffraction peaks decreased with the increasing hydrothermal time, implying the decreased crystallinity of samples. Secondly, the broadening of the diffraction peaks also be observed, which may be due to the combined effect of decreasing grain size and increasing microstrains [27,28,29]. Table 1 displays the crystallite sizes (d_XRD_) estimated from the half-band width of the corresponding X-ray spectral peak by the Scherrer Formula [30]. As the hydrothermal time increases, the grain size of TiO_2_ gradually decreases. This may be due to the alkali ions penetrated into TiO_2_ lattice, or they probably are bonded with oxygen onto TiO_2_ surface to form alkali metal oxides, both of which restricted the growth of TiO_2_ nanocrystals [31]. TiO_2_-48 possesses the smallest crystallite size calculated by Scherrer Formula, however, the specific surface area of TiO_2_-48 is not the largest. The contradiction arises because the morphology of TiO_2_-48 has changed and the calculation results of XRD only represent the size of the (101) crystal plane instead of the true size of TiO_2_-48 [32,33]. 

The anatase phase of the TiO_2_ samples before and after hydrothermal process is further confirmed by the typical Raman bands at 138 cm^−1^ (E_g_), 191 cm^−1^ (E_g_), 391 cm^−1^ (B_1g_), 510 cm^−1^ (A_1g_) and 634 cm^−1^ (E_g_) in Figure S1 [34]. Notably, the intensity of the peaks become weaker after hydrothermal treatment, possibly due to the poorer crystallinity [35]. It is reported that the higher the crystallinity, the fewer defects in the crystal. Therefore, defects (oxygen vacancies) may exist in TiO_2_-X samples after hydrothermal treatment [36]. Moreover, compared with the pristine the TiO_2_, low frequency peak at 138 cm ^−1^ (E_g_) of the TiO_2_-X samples after hydrothermal treatment exhibits slight blue shift and significant broadening. And the E_g_ peak of TiO_2_-24 is particularly prominent. It shifts from 137.82 cm^−1^ to 140.07 cm^−1^ and the full width at half maximum (FWHM) increases from 8.29 to 12.65. Generally, the quantum size limitation effect (that is to say decreased crystalline size) causes red shift and broadened peak while the presence of defects cause blue shift [37,38]. This result shows the effect of defects are dominant compared to crystalline size. To sum up, the variations of Raman shift and FWHM are the combined effect of crystalline size and defects.

**Figure 3:**
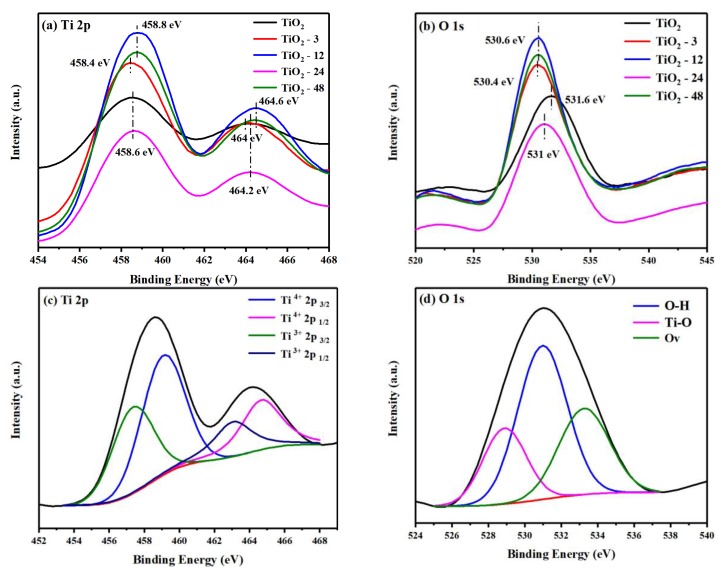
XPS spectra of the (**a**) Ti 2p band of TiO_2_, TiO_2_-3, TiO_2_-12, TiO_2_-24, TiO_2_-48, (**b**) O 1s band of TiO_2_, TiO_2_-3, TiO_2_-12, TiO_2_-24, TiO_2_-48, (**c**) Ti 2p band of TiO_2_-24 and (**d**) O 1s band of TiO_2_-24.

The surface component and chemical valence state of TiO_2_-X are further investigated by XPS spectra. The high-resolution Ti 2p XPS spectrum of pristine TiO_2_ and TiO_2_-24 are compared. In Figure S2a, the fitting result of pristine TiO_2_ shows two peaks at 457.2 eV and 463.0 eV, this corresponds to the Ti^3+^ 2p_3/2_ and Ti^3+^ 2p_1/2_, indicating that Ti^3+^ already exists in pristine TiO_2_. Similarly, Ti^3+^ 2p_3/2_ and Ti^3+^ 2p_1/2_ peaks can also been detected in Figure 3c, certifying the existence of Ti^3+^ in addition to the Ti^4+^ oxidation state for TiO_2_-24 samples [39]. 

According to the peak areas, the Ti^3+^/Ti^4+^ ratio of TiO_2_-24 was calculated to be 85.9%, while the Ti^3+^/Ti^4+^ ratio of pristine TiO_2_ was 67.2% (Table S2). Usually, Ti^4+^ could be reduced to Ti^3+^ via trapping electron from oxygen vacancy, so the increased Ti^3+^/Ti^4+^ ratio was able to verify indirectly the existence of surface oxygen vacancies [40,41]. The high-resolution O 1s XPS spectrum of pristine TiO_2_ and TiO_2_-24 both be fitted into three peaks of around 529 eV, 531 eV and 533 eV, which are ascribed to lattice oxygen (Ti-O), surface hydroxyl groups (-OH) and the oxygen vacancies (O_v_) in the vicinity of Ti^3+^ [42]. Compared with the pristine TiO_2_, the high-resolution O 1s XPS spectrum of TiO_2_-X shifted to a lower energy because of the band bending effect caused by extra electrons from oxygen vacancies on TiO_2_ crystal lattice or to satisfy the requirement of charge equilibrium [34,43]. Ti^3+^ and oxygen vacancies can serve as photoinduced charge traps as well as adsorption sites to prevent the electron-hole recombination, which are conducive to the improving photocatalytic activity [44,45]. 

**Figure 4:**
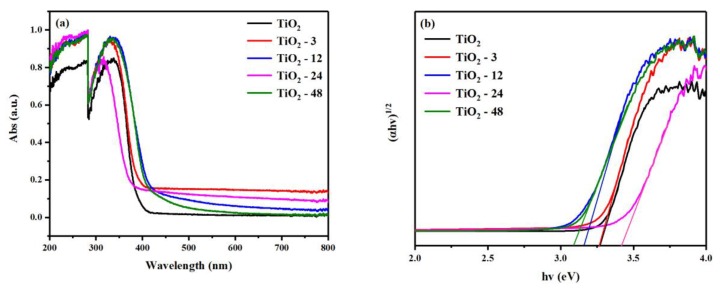
UV-Vis diffuse reflectance spectra (**a**) and bandgap energy (**b**) of TiO_2_-X.

Ultraviolet-visible diffuse reflectance spectroscopy (DRS) is a common method to characterize the optical absorption properties of materials. As displayed in Figure 4a, the pristine TiO_2_ shows a strong absorption in ultraviolet region and the absorption edge is located at 400 nm, which is consistent with the light absorption characteristics of anatase TiO_2_ [46]. For TiO_2_-X, it exhibits increased absorption in the region of 200–800 nm after hydrothermal treatment, which can be attributed to surface oxygen vacancies and Ti^3+^ [44,47]. According to the Kubelka-Munk function, the band gap energies can be calculated from the plots of (αhv)^1/2^ versus photon energy [43]. The band gap energies of pristine TiO_2_, TiO_2_-3, TiO_2_-12, TiO_2_-24 and TiO_2_-48 are 3.27 eV, 3.26 eV, 3.16 eV, 3.42 eV and 3.09 eV, respectively (Table 1). The wider the band gap, the stronger the redox capability of the photocatalysts. The enhanced visible light absorption is attributed to the presence of surface oxygen vacancies and Ti^3+^, which could form a shallow donor lever just below the conduction band, thus narrowing the band gap and response to visible light [32].

### 3.2. Evaluation of the Photocatalytic Efficiency

**Figure 5:**
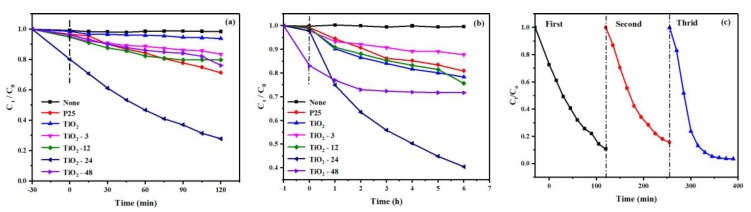
Photocatalytic degradation of Rhodamine B (**a**) and Sulfadiazine (**b**) over different photocatalysts under visible light irradiation; (**c**) three-cycle photocatalytic degradation of Rhodamine B over TiO_2_-24.

Photocatalytic performance of TiO_2_-X samples was evaluated by degradation of RhB and SD under visible light. Pristine TiO_2_, P25 and the pollutants without photocatalyst were used as control samples. P25 is a commercially produced titanium dioxide material with high photocatalytic activity and is often used as a benchmark photocatalyst [48]. The control experiment in Figure S3 showed that all SD samples reach the adsorption/desorption equilibrium in the dark already after 1 hour. In Figure 5a,b, All TiO_2_-X samples exhibited better photocatalytic performance under visible light than pristine TiO_2_ for RhB and SD degradation. The TiO_2_-24 showed the best performance, approximately 72.7% of RhB was degraded within 120 min and 59.5% of SD was degraded within 6 hours. For pristine TiO_2_, TiO_2_-3, TiO_2_-12 and TiO_2_-48, the degradation efficiency of RhB exhibited 6.2%, 16.5%, 20.4%, 23.9% within 120 min and the degradation efficiency of SD was 21.7%, 12.2%, 24.3%, 28.3% within 6 hours, respectively. The photodegradation curves of RhB and SD were fitted by pseudo-first-order reaction kinetics. The rate constant (k) of degrading RhB (Figure S4) was calculated to be 0.0088 min^−1^ for the TiO_2_-24 and 0.00258 min^−1^ for P25, and the k of degrading SD was calculated to be 0.13931 h^−1^ for the TiO_2_-24 and 0.03303 h^−1^ for P25. Whether degrading RhB or SD, the degradation rate constant of TiO_2_-24 is much higher than P25, and the degradation rate constant of TiO_2_-24 is about 4 times that of P25. The degradation performance of SD is consistent with the degradation trend of RhB. This indicates that TiO_2_ samples treated by alkaline hydrothermal method can be used as a useful photocatalyst for both dyes and likely also for other organic pollutants. This also demonstrates that the morphology of the nanomaterial has a certain influence on its photocatalytic effect.

In addition to the high photocatalytic activity of catalysts, stable recyclability is also essential for their practical application. In Figure 5c, the photocatalytic stability of TiO_2_-24 was investigated by cyclic photocatalytic degradation of RhB solution. No obvious reduction was observed after three cycles. It is indicated that TiO_2_-24 exhibited relatively high photodegradation performance after a long time irradiation.

### 3.3. The mechanism of Photocatalytic Performance Improvement of the TiO_2_-X.

**Figure 6:**
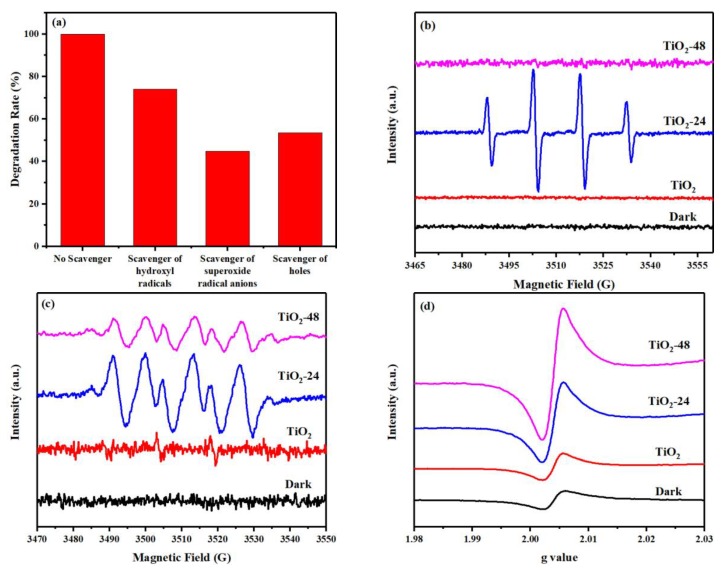
(**a**) Free radical trapping test of hydroxyl radicals, superoxide radicals and holes over TiO_2_-24; Electron spin resonance spectra of radical in TiO_2_-24 under visible light: (**b**) DMPO-·OH and (**c**) DMPO-O_2_^•−^ (**d**) h^+^.

The trapping experiments were carried out to investigate the main reactive oxygen species (ROS) generated in the photocatalytic degradation process. EDTA-2Na, tert-butyl alcohol (t-BuOH) and N_2_ were used as holes (h^+^) scavenger, hydroxyl radicals (·OH) scavenger and superoxide radicals (O_2_^•-^) scavenger, respectively [49]. Figure 6a suggests the degradation of RhB over TiO_2_-24 was suppressed by the addition of the three scavenger, indicating that h^+^, ·OH and O_2_^•-^ might be the active species on the RhB degradation process, especially O_2_^•-^. This phenomenon may be caused by the efficient photogenerated electron transfer in the TiO_2_-24 nanoparticles, which facilitates more O_2_^•-^ participation in the photodegradation of RhB. 

To further confirm the radicals generated in the reaction process of TiO_2_-24, ESR analysis was also performed using DMPO as the radical spin-trapping probe [50,51]. After illuminating with visible light for 10 min, TiO_2_-24 exhibits the strongest intensity of DMPO-O_2_^•−^ signals, indicating that the amounts of O_2_^•−^ produced in TiO_2_-24 is much larger than that for pristine TiO_2_ and TiO_2_-48, which is consistent with the photodegradation performance.

ESR can also be used to confirm the presence of oxygen vacancies. As shown in Figure 6d, there is a sharp signal at g = 2.004, which is identified as the electrons trapped on surface oxygen vacancies. Pristine TiO_2_, TiO_2_-24 and TiO_2_-48 exhibited the enhanced signal intensity of oxygen vacancies after illuminating 10 min under visible light, indicating that the oxygen vacancies were also involved in the degradation process under visible irradiation [52,53]. The single intensity increased from TiO_2_ to TiO_2_-48, indicating the surface oxygen vacancies concentrations of TiO_2_ increased with extending hydrothermal time [54]. However, when the surface oxygen vacancies concentration is too high, bulk oxygen vacancies can easily be generated [9]. The crystalline performance decreases, the bulk defects increases, the results of XRD also verified the existence of bulk defects in TiO_2_-48. Surface oxygen defects can serve as photoinduced charge traps as well as adsorption sites where the charge transfer to adsorbed species can prevent the electron-hole recombination, which is important for improving of photocatalytic activity; whereas bulk oxygen vacancies only act as charge traps where electron-hole recombines, resulting in the decrease of photoactivity [43,55]. 

### 3.4. Photoinduced Electron Transfer Properties in the TiO_2_-X.

**Figure 7:**
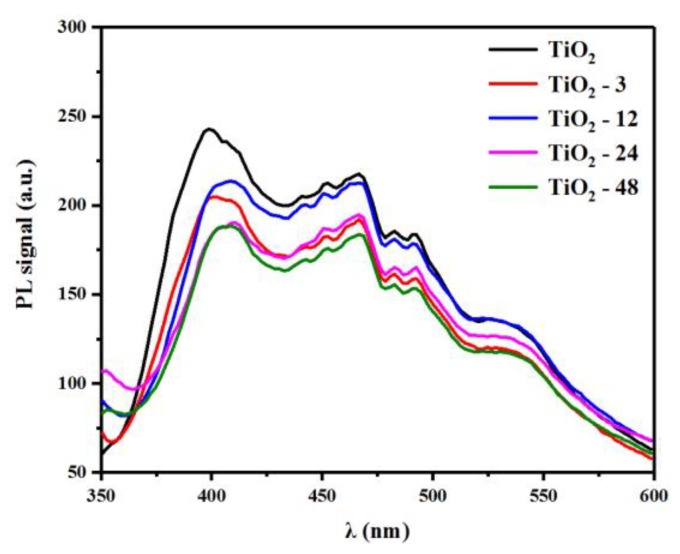
Photoluminescence spectra of TiO_2_-X.

Photoluminescence (PL) spectra is a useful technique to investigate the oxygen vacancies through the measurement of the charge carrier trapping, immigration and recombination [56]. In Figure 7, the highest intensity emission peak around 433 nm corresponds to the emission of self-trapped excitons located on TiO_6_, which is consistent with the position of absorption edge band in DRS [57,58]. The peaks in the range of 450–500 nm are originated from surface oxygen vacancies and Ti^3+^ [59,60]. The peaks at around 530 nm are derived from the oxygen vacancies buried in the bulk of materials [61]. The surface defects can form the exciton energy level near the bottom of the conduction band, which allows them to act as trapping sites and hinders the recombination of photogenerated chargers [36,62]. In Figure 7, the signals of TiO_2_-X are similar, but the peak intensity changed obviously. According to XRD, XPS and ESR results, the pristine TiO_2_ possesses good crystallinity as well as little defects. After alkali-hydrothermal treatment, the decreased crystallinity of TiO_2_-X is accompanied by the increased defects, which is beneficial to hinder the recombination of photogenerated chargers, thus the emission peak intensity of TiO_2_-X is weakened [35,63]. However, TiO_2_-48 possesses an excess of surface defects and forms bulk defects, thus the peak intensity was higher than TiO_2_-24.

**Scheme 1:**
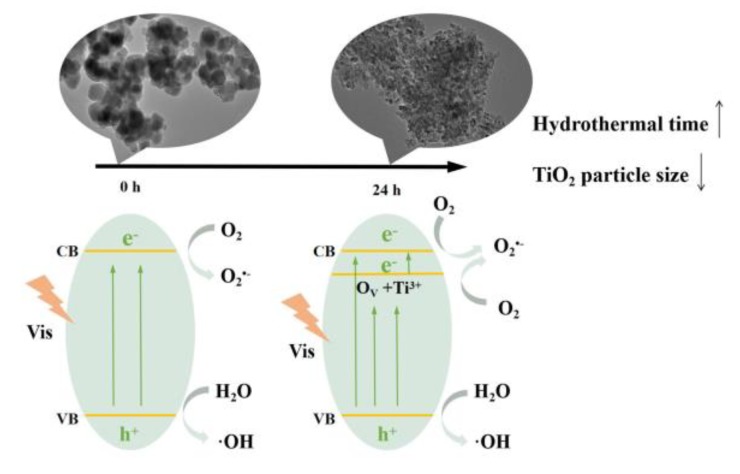
Schematic image of different sized TiO_2_ nanoparticles.

On the basis of the above results, the surface defects and crystalline sizes played a dominating role on photocatalytic performance. Scheme 1 is the photocatalytic process of TiO_2_-24 with Ti^3+^ and oxygen vacancies (O_v_). For pristine TiO_2_, the electrons are injected from its valence band to the conduction band under visible light irradiation. For TiO_2_-24, the Ti^3+^ and O_v_ form local state below the conduction band, and the electrons are promoted from the valance band to Ti^3+^ and O_v_ state under visible light irradiation. The electrons on Ti^3+^ and O_v_ state cannot recombine easily with photogenerated holes as the Ti^3+^ and O_v_ are electron traps. Then the electrons react with the surface-absorbed O_2_ to generate active oxygen radicals O_2_^•-^. The larger specific surface areas creates more basic sites benefit for the increased adsorption of the pollutant molecules. The existence of Ti^3+^ and O_v_ accelerate the electron transfer rate, and suppress the photogenerated e^-^-h^+^ recombination. As a consequence, TiO_2_-24 showed the enhanced photocatalytic performance.

## 4. Conclusions

The TiO_2_ samples were modified at different hydrothermal times (0–48 h) with a low alkali-hydrothermal method. The physicochemical properties and photocatalytic performance of the prepared TiO_2_-X nanoparticles with different crystalline sizes were explored. With the increased time of hydrothermal treatment, the crystalline size of TiO_2_-X decreased first and then increased, and the crystallinity decreased. An annealing in N_2_ atmosphere after hydrothermal treatment caused TiO_2_ reduction and created defect. The appropriate concentration of surface oxygen vacancies is beneficial to separate photogenerated charges. Due to the combined effects of crystalline size and surfaces defects, the efficiency of photocatalytic degradation of SD and RhB by TiO_2_-24 nanoparticle under visible light was obviously improved. This study highlights the effects of particle size and surface oxygen defects on the structure and performance of photocatalysts. It opens a new window for the purification of water environmental pollution. 

## Figures and Tables

**Table 1:** The specific surface area (S_BET_), pore size and pore volume, band gap (E_g_) and crystalline size (d_XRD_) of TiO_2_-X.

Sample	S_BET_^1^ (m^2^/g)	Pore Volume ^2^ (cm^3^/g)	Pore Diameter ^2^ (nm)	E_g_^3^ (eV)	d_XRD_^4^ (nm)
TiO_2_	13.060	0.035	1.335	3.27	12.96
TiO_2_-3	23.164	0.099	1.149	3.26	7.92
TiO_2_-12	59.742	0.102	1.336	3.16	3.94
TiO_2_-24	78.888	0.312	1.145	3.42	3.44
TiO_2_-48	1.592	0.002	2.592	3.09	3.04

^1^ The specific surface area (S_BET_) was calculated by a Brunauer-Emmett-Teller (BET) method using the adsorption data in relative pressure (P/P_0_) range from 0.04 to 0.3. ^2^ Pore volume and pore diameter were calculated by nitrogen adsorption volume at P/P_0_ = 0.99 using Barrett-Joyner-Halenda (BJH) method. ^3^ The band gap energies (E_g_) can be calculated from the plots of (αhv)^1/2^ versus photon energy. ^4^ Average crystalline size (d_XRD_) of TiO_2_ was calculated by the anatase (101) plane of XRD using the Scherrer equation.

## Supplementary Materials

The following are available online at https://www.mdpi.com/2079-4991/10/3/546/s1, Figure S1: Raman spectra of TiO_2_-X, inset is an enlarged view of TiO_2_-X in the 300–700 cm^−1^ region. Figure S2: XPS spectra of the (a) Ti 2p band of pristine TiO_2_, (b) O 1s band of pristine TiO_2_. Table S2: Ratio of peak areas of Ti^3+^ and Ti^4+^ (Ti^3+^/Ti^4+^) according to the quantitative analyses of the fitted Ti 2p XPS peaks. Figure S3: The adsorption test of Sulfadiazine. Figure S4. (a) Rhodamine B photodegradation kinetics of P25 and TiO_2_-X, (b) Sulfadiazine photodegradation kinetics of P25 and TiO_2_-X, (c) RhB photodegradation rate constants k of P25 and TiO_2_-X and (d) SD photodegradation rate constants k of P25 and TiO_2_-X.
