# Supplementary material for: Effects of Particle Size on the Structure and Photocatalytic Performance by Alkali-Treated TiO2
Source: Nanomaterials (Basel). 2020 Mar 18;10(3):546. doi: 10.3390/nano10030546 (PMC7153365; doi:10.3390/nano10030546)
Supplement: Supplementary file 1 [file nanomaterials-10-00546-s001.zip › nanomaterials-737335-supplementary.doc]

***Supporting Information for Nanomaterials***

Article

Effects of Particle Size on the Structure and Photocatalytic Performance by Alkali-Treated Tio_2_

Danqi Li ^1, †^, Hongchen Song ^1, †^, Xia Meng ^1^, Tingting Shen^1^, Jing Sun ^1,^*, Wenjia Han^1^ and Xikui Wang ^2,^*

^1^	State Key Laboratory of Biobased Material and Green Papermaking, School of Environmental Science and Engineering, Qilu University of Technology (Shandong Academy of Sciences), Jinan 250353, P.R. China; autantenporte@163.com (D. L.); shc01261005lx@gmail.com (H.S.); mengxia7184@126.com (X.M.); [stthunanyt@163.com](mailto:stthunanyt@163.com) (T.S.); hwj200506@163.com (W.H.)

^2^	College of Environmental Science and Engineering, Shandong Agriculture and Engineering University, Jinan 251100, P.R. China.

*****	Correspondence: jingsunok@163.com (J.S.); xk_wang@qlu.edu.cn (X.W.)

†	These authors contributed equally to the work.

Received: 19 February 2020; Accepted: 13 March 2020; Published: March 2020

(2 Page, 4 Figures, 1 Table)

**Figure S1.** Raman spectra of TiO_2_-X, inset is an enlarged view of TiO_2_-X in the 300–700 cm^-1^ region.

|  |  |
| --- | --- |

**Figure S2.** XPS spectra of the (**a**) Ti 2p band of pristine TiO_2_, (**b**) O 1s band of pristine TiO_2_.

**Table S2.** Ratio of peak areas of Ti^3+^ and Ti^4+^ (Ti^3+^/Ti^4+^) according to the quantitative analyses of the fitted Ti 2p XPS peaks.

| **Sample** | **TiO_2_** | **TiO_2_-3** | **TiO_2_-12** | **TiO_2_-24** | **TiO_2_-48** |
| --- | --- | --- | --- | --- | --- |
| **Ti^3+^/Ti^4+^ (%)** | 67.2 | 70.9 | 76.3 | 85.9 | 92.0 |

**Figure S3.** The adsorption test of Sulfadiazine.

|  |  |
| --- | --- |
|  |  |

**Figure S4.** (**a**) Rhodamine B photodegradation kinetics of P25 and TiO_2_-X, (**b**) Sulfadiazine photodegradation kinetics of P25 and TiO_2_-X, (**c**) RhB photodegradation rate constants k of P25 and TiO_2_-X and (**d**) SD photodegradation rate constants k of P25 and TiO_2_-X.
